# Supplementary material for: Mitochondrial PCK2 Missense Variant in Shetland Sheepdogs with Paroxysmal Exercise-Induced Dyskinesia (PED)
Source: Genes (Basel). 2020 Jul 9;11(7):774. doi: 10.3390/genes11070774 (PMC7397061; doi:10.3390/genes11070774)
Supplement: Supplementary file 1 [file genes-11-00774-s001.zip › FigureS1.pdf]

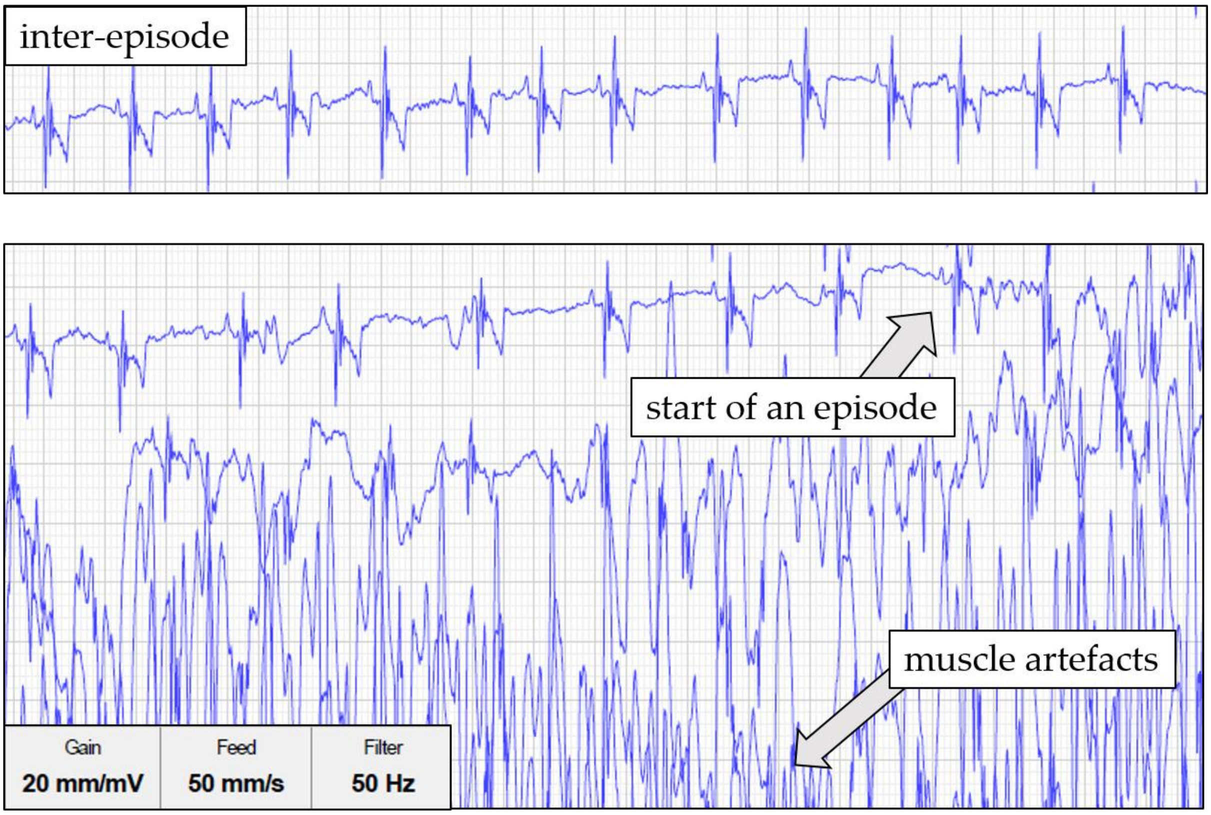

Figure S1. Long-term electrocardiography (ECG) of case 4 before a dyskinetic episode and at the beginning of an episode. ECG between and before an episode reveals normal sinus rhythm with mild tachycardia (120-180 bpm). When a dyskinetic episode starts, ECG is overlapped by muscle artefacts. ECG did not reveal cardiac pathology.
